# Coupling Genetic and Chemical Microbiome Profiling Reveals Heterogeneity of Archaeome and Bacteriome in Subsurface Biofilms That Are Dominated by the Same Archaeal Species

**DOI:** 10.1371/journal.pone.0099801

**Published:** 2014-06-27

**Authors:** Alexander J. Probst, Giovanni Birarda, Hoi-Ying N. Holman, Todd Z. DeSantis, Gerhard Wanner, Gary L. Andersen, Alexandra K. Perras, Sandra Meck, Jörg Völkel, Hans A. Bechtel, Reinhard Wirth, Christine Moissl-Eichinger

**Affiliations:** 1 Institute for Microbiology and Archaea Center, University of Regensburg, Regensburg, Germany; 2 Center for Environmental Biotechnology, Lawrence Berkeley National Laboratory, Berkeley, California, United States of America; 3 Department for Bioinformatics, Second Genome Inc., South San Francisco, California, United States of America; 4 Department of Biology I, Biozentrum, LMU Munich, Planegg-Martinsried, Germany; 5 Department of Geomorphology and Soil Science, Technische Universität München, Center of Life and Food Sciences Weihenstephan, Freising, Germany; 6 Advanced Light Source, Lawrence Berkeley National Laboratory, Berkeley, California, United States of America; Graz University of Technology (TU Graz), Austria

## Abstract

Earth harbors an enormous portion of subsurface microbial life, whose microbiome flux across geographical locations remains mainly unexplored due to difficult access to samples. Here, we investigated the microbiome relatedness of subsurface biofilms of two sulfidic springs in southeast Germany that have similar physical and chemical parameters and are fed by one deep groundwater current. Due to their unique hydrogeological setting these springs provide accessible windows to subsurface biofilms dominated by the same uncultivated archaeal species, called SM1 Euryarchaeon. Comparative analysis of infrared imaging spectra demonstrated great variations in archaeal membrane composition between biofilms of the two springs, suggesting different SM1 euryarchaeal strains of the same species at both aquifer outlets. This strain variation was supported by ultrastructural and metagenomic analyses of the archaeal biofilms, which included intergenic spacer region sequencing of the rRNA gene operon. At 16S rRNA gene level, PhyloChip G3 DNA microarray detected similar biofilm communities for archaea, but site-specific communities for bacteria. Both biofilms showed an enrichment of different deltaproteobacterial operational taxonomic units, whose families were, however, congruent as were their lipid spectra. Consequently, the function of the major proportion of the bacteriome appeared to be conserved across the geographic locations studied, which was confirmed by *dsrB*-directed quantitative PCR. Consequently, microbiome differences of these subsurface biofilms exist at subtle nuances for archaea (strain level variation) and at higher taxonomic levels for predominant bacteria without a substantial perturbation in bacteriome function. The results of this communication provide deep insight into the dynamics of subsurface microbial life and warrant its future investigation with regard to metabolic and genomic analyses.

## Introduction

The subsurface biosphere harbors an enormous portion of the Earth's microbiome. It is estimated, that approximately 2.9×10^29^ and 2.5–25×10^29^ prokaryotic cells reside below the surface layer in marine and terrestrial sediments, respectively [1], [2]. Sampling, and thus exploration of the subsurface microbiomes by deep drilling is difficult, since each sample is subject to a possible contamination by surface microorganisms [1]. Currently, subsurface biotopes remain poorly understood as microbial and biogeochemical “dark matter” but have substantial contribution to carbon, nitrogen and sulfur cycling [1], [3], [4]. However, important windows to the subsurface are provided by (artesian) aquifers and their natural and artificial springs [4], [5], delivering possibly 10^3^–10^6^ prokaryotic cells/ml to the surface [1], [6]. Although sulfidic springs are rather rare (10% of all terrestrial aquifers; [7]), they contain excellent energy sources for subsurface and also surface life: Once mixed with oxygen as terminal electron acceptor nutrients from sulfidic subsurface aquifers can lead to high amounts of biomass in the outflow region [8]–[14]. These biomasses, which are mostly complex microbial communities such as bacterial or archaeal/bacterial biofilms, have been the focus of many studies, yet the oxygen-free subsurface environment of sulfidic springs is lacking information concerning its biodiversity and variation over geographical location [15].

**Figure 1:**
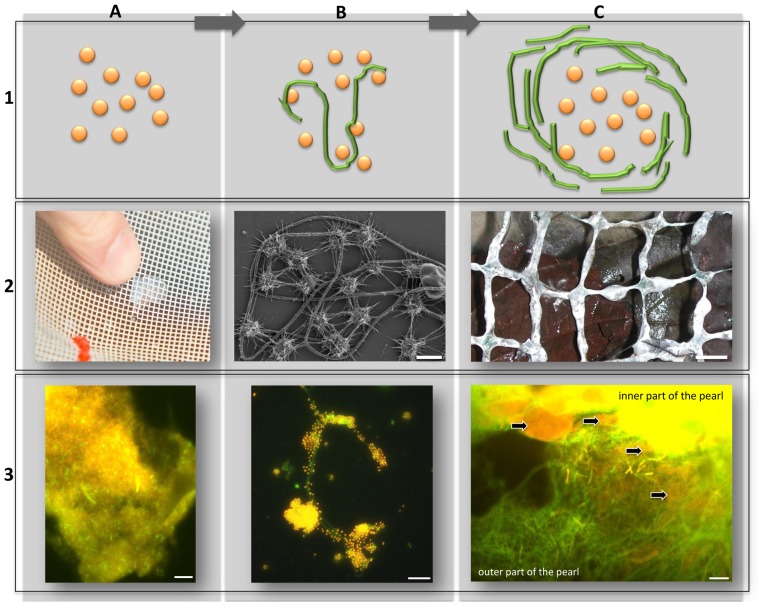
The conversion of biofilm to string-of-pearls community in the spring water originating from the subsurface. A: Biofilm. B: Intermediate transition state. C: String-of-pearls community. Row 1: Schematic drawings. Orange: SM1 euryarchaeal cocci, Green: Filamentous, sulfide-oxidizing bacteria. Row 2: Photographs and scanning electron micrograph (2B) of different stages. Row 3: FISH images of different stages (for MSI samples please see [15]; Archaea orange (CY3), Bacteria green (RG)). A: SM-BF, showing high dominance of Archaea. B: Attachment of archaea to filamentous bacteria. C: String-of-pearls communities with large archaeal colony and bacterial mantle. Arrows point to archaeal microcolonies, manteled by filamentous bacteria. It is proposed that attachment of SM1 Euryarchaeota to filamentous bacteria (B) mediates the transition from biofilm (A) to the string-of-pearls community (C). Scale bars: A3: 10 µm, B2: 1 µm B3: 10 mm, C3: 25 µm.

In southeast Germany near Regensburg, sulfur springs rise out of the subsurface karst system in the Jurassic carbonate settings. Due to the hydrogeological conditions, atmospheric oxygen mixed with the cold (∼10°C), anoxic sulfidic groundwater leads to a sudden increase in biomass and the appearance of the microbial “string-of-pearls community”, observed in two distinct spring areas, the Sippenauer Moor and the Mühlbacher Schwefelquelle. In the “pearls”, the uncultivated, phylogenetically deep-branching SM1 Euryarchaeon resides, surrounded by sulfur-oxidizing bacteria (*Thiothrix* sp., site Sippenauer Moor, [11], [14], for graphical illustration the reader is referred to Fig. 1). This specific partnership of microbial members of two domains of life is highly specific and stable, and a syntrophic interaction is hypothesized [16]. Although an inter-species sulfur-cycle between the archaea and the bacteria (sulfur-oxidizers) had been proposed [11], no evidence for sulfate-reduction by the SM1 Euryarchaeon could be collected during a recent study [15]. A detailed characterization of the archaeal/bacterial community revealed a remarkable trait of the SM1 Euryarchaeon: the *hami* (singular *hamus*). These cell surface appendages are on average 2 µm long, occur as hundreds per cell and consist of a barbwire structure (“prickle region”) with a nano-grappling hook at the distal end [17]. The *hami* are a unique feature of the SM1 Euryarchaeon and are considered a potential biomarker since they have never been observed for any other organism.

In the subsurface of the Mühlbacher Schwefelquelle, the SM1 Euryarchaeon was found to form an almost pure biofilm [18], consisting of a dense network of cells mediated by the *hami*. Such biofilm pieces are constantly washed up from deeper Earth layers and can be harvested at the spring outflow. In the biofilm, the archaea are associated with a minor bacteriome, dominated by sulfate-reducing bacteria [15]. The constant predominance of the SM1 Euryarchaeon (>95%) in the subsurface biofilms was demonstrated by different methods and in hundreds of samples taken between 2005 and 2013 from the Mühlbacher Schwefelquelle [15], [18]. Minor investigations also included samples from the Sippenauer Moor, where the appearance of these subsurface biofilms was also observed but not further documented [18]. Both springs delivering these biofilms provide a window to subsurface biotopes and the microbial communities are a model system for cold-loving and biofilm-forming archaea as well as for subsurface research in general [15]. What is missing in the current body of literature is a comparative study on the biofilms that can be harvested from the two different sampling sites. Such a comparative analysis could shed light onto the biodiversity variation of microbial subsurface life across the geographic locations, which are suggested to be supplied by the same deep waterflow based on their hydrogeology.

In this study, we investigated microbial differences of subsurface biofilm samples from both sampling sites and specifically focused on the variation in the microbial composition, biochemical properties, the cell surface ultrastructure and fingerprinting of *hamus* gene occurrence [17]. Empirically determined operational taxonomic units (eOTUs) derived from PhyloChip G3 data were used for microbial community profiling at 16S rRNA gene level. To add an extra dimension to the knowledge of the biochemistry of the SM1 Euryarchaeon biofilms, we applied multivariate statistics to the chemical imaging data acquired by means of synchrotron radiation-based Fourier transform infrared (SR-FTIR) spectromicroscopy.

## Materials and Methods

### The hydrogeology of Sippenauer Moor (SM) and Mühlbacher Schwefelquelle (MSI)

The sulfur springs at the Sippenauer Moor (SM) near Kelheim (Lower Bavaria) rise out of the subsurface karst system developed in the Jurassic carbonate setting [19]. To the contrary, the Mühlbacher Schwefelquelle at Isling (MSI) near Regensburg (Upper Palatonia) is not a natural spring (map in Fig. S1). It is a well drilled to a depth of 36.5 m in the year 1925, but has never been used for drinking water supply because of a strong sulfur odor (information provided by REWAG Regensburger Energie- und Wasserversorgung AG & Co KG, the electricity and water supply institute at Regensburg, Germany). The MSI site is situated in the transition area of a river terrace of the Danube from pre-Eemian times (Riss glaciation), covered with Wuermian Loess and Loess Loam. The drill log from 1925 notes the 2.6 m thick Loess layer, the interlayering fluvial sediments and the Quaternary base, which overlies the top of the sedimentary Cretaceous bedrock (“Regensburger Grünsandstein”). At a depth of 23.45 m, the well reached an artesian groundwater table (aquifer) with strong discharge and sulfuric odor. It has to be assumed that the well reached the stratigraphic boundary between Cretaceous and Jurassic sediments (Malm), which are both described as (calciferous) sandstones.

The springs at both locations are connected to the deep water flow within the pre-alpine Tertiary Molasse basin [20]. The deep aquifer is developed in the karst and fracture system of the underlying Jurassic sediments. Stable isotope geochemistry at comparable sites within the region (Lower Bavaria) revealed the constant drainage of pore water from the hanging Molasse and Cretaceous layers into the underlying karstified Jurassic layers [21]. Isotope mixing ratios that have remained unchanged for decades and one radiocarbon age (^14^C) of 30,000 years (uncalibrated) revealed the long term runoff of water within the deep Malm karst system within the Molasse basin towards the Danube valley downstream of Regensburg [21].

Even though the sources for hydrogen sulfide of sulfur springs elsewhere in Lower and Upper Bavaria are bituminous Mesozoic sediments, pyrite rich Jurassic sediments (Lias) or Tertiary brown coal deposits [22], [23], the sulfide at SM and MSI comes from microbial sulfate reduction of substances set free out of salinar formations (Zechstein) located at the alpine rim of the Molasse basin [23]. Inorganic reduction processes can be excluded because of the lack of higher temperatures [23].

#### Sample collection

The sampling permit for the Sippenauer Moor was issued by the Regensburgische Botanische Gesellschaft von 1790 e.V., Regensburg. The sampling permit for the Mühlbacher Schwefelquelle was obtained from Gartenamt Regensburg. The field studies did not involve endangered or protected species. Concentrations of H_2_S and dissolved oxygen in the spring waters were measured using a colorimetric hydrogensulfide test (Merck KG, Darmstadt, Germany; [13]) or using a highly sensitive oxygen dipping probe (PreSens, Regensburg, Germany; [15]).

Samples from the Sippenauer Moor were collected by polyethylene nets. These nets were either used to filter water directly at the spring outlet in order to harvest milky, slimy biofilms washed up from deeper earth layers or to provide attachment and growth conditions for string-of-pearls community at 0.65 m to 0.80 m distance from the spring (under oxygen-enriched conditions). The collected samples included three Sippenauer Moor biofilm (SM-BF) samples taken directly from the spring outflow where the oxygen and H_2_S concentrations were 0.02 mg/l and 0.85 mg/l, respectively, and six string-of-pearls community (SOPC) samples taken at a location where the outflows of three springs mix (oxygen concentrations were 0.89 and 1.10 mg/l respectively; sulfide concentration at both areas: 0.5 mg/l).

The second sampling location was the Mühlbacher Schwefelquelle nearby Isling (MSI; linear distance 20 km to Sippenauer Moor; N48°59.140′E12°07.631′; [13] [18]; Fig. S1). Three MSI biofilm (MSI-BF) samples were harvested in a similar manner under oxygen-free conditions (samples from [15]); right at the sampling area (within the spring hole), the H_2_S concentration was 0.85 mg/l and the oxygen concentration was below detection limit (details see: [15]).

The chemical water composition of the two springs was documented earlier [13] and showed high similarity in most chemical parameters measured, including compounds such as Fe, B, K^+^, Mg^2+^, Ca^2+^, Cl^−^, Na^+^ and S_2_O_3_^−^ (Table S1). However, water from the Mühlbacher Schwefelquelle revealed slightly higher concentrations in CO_2_, ammonia and sulfide, whereas SM water contained higher concentrations of sulfate (Table S1).

#### Sample preparation

Biofilm samples were removed from the polyethylene nets with syringes/pipettes and transported to the laboratory on ice. Samples for DNA extraction were stored at −20°C, and samples for SR-FTIR analyses were air-dried on gold-coated grids after removing the liquid [15]. Samples for whole-cell fluorescence *in situ* hybridization (FISH) were prepared as described earlier [14].

#### DNA extraction and quantitative PCR (qPCR)

DNA was extracted from samples using the XS-buffer protocol [24]. Bacterial and archaeal 16S rRNA genes as well as *dsrB* (dissimilatory sulfite-reductase subunit B) genes were quantified by qPCR as described previously [15], [24]. For each sample type (MSI biofilm, SM biofilm, SOPC) three independent biological replicates were individually measured three times.

#### Amplicon generation for microarray analysis [15]

Here, bacterial 16S rRNA genes were amplified from metagenomic DNA samples with primer pair 27F and 1492R [25], whereas archaeal 16S rRNA gene with 345af and 1406ur [26], [27]. Amplicons were purified by agarose-gel electrophoresis as performed earlier [15]. All 15 samples for microarray analyses were taken within one day, PCR amplified and moved forward for microarray hybridization: 3× MSI-BF, 3× SM-BF and 6× SOPC, 1× MSI water, 1× SM water, and 1× extraction blank. Water samples and extraction blank were used for control purposes.

#### PhyloChip G3 data acquisition

The PhyloChip G3 Assay (Second Genome, South San Francisco, CA) and analysis were carried out as described earlier [25]. Briefly, bacterial (500 ng) and archaeal (100 ng) 16S rRNA gene amplicons were combined, spiked with a known amount of non-16S rRNA genes for standardization, fragmented and biotin labeled. After hybridization on DNA microarrays, images were scanned, background and noise was determined, and fluorescence intensity was scaled to the spike-in internal controls [25].

#### Empirical OTU (eOTU) discovery from PhyloChip data [28]

The 25-mer 16S rRNA gene probes were compared to their mismatch controls [25] and 24,154 were found to be responsive in at least three microarray datasets (biological samples). Taxonomically related probes were clustered into probe-sets where pair-wise correlations ≥0.85 between log_2_ transformed fluorescence intensities (FI) were discovered as described by Probst et al. 2014 [28]. A total of 1380 probe-sets were found and the empirical operational taxonomic units (eOTU) tracked by each probe-set were taxonomically annotated against the 2012 taxonomy using a Naive Bayesian scoring and >80% bootstrapped confidence cutoff [29], [30]. The mean log_2_ FI among the multiple probes for each eOTU was calculated for each sample. These values are referred to as the hybridization score (HybScore) used in PhyloChip abundance-based analysis. For details please see Supplementary Information and Probst et al. 2014 [28]. 32 eOTUs detected in the DNA extraction blank were removed from further analyses as were those 12 eOTUs only present in spring water samples, resulting in 1337 eOTU considered for microbiome analyses. PhyloChip eOTU data was made publicly available at http://greengenes.secondgenome.com/downloads/phylochip_datasets along with the raw data (CEL files, filename Probst_2014_aquifer.tar.gz).

#### Statistical analysis of microarray data

Second Genome's Microbial Profiling Analysis Pipeline (PhyCA-Stats™) was used for univariate and multivariate statistics of abundance scores (hybridization scores) of all eOTUs that were called present in at least one of the samples. The analyses included hierarchical clustering (average neighbor), NMDS (non-metric multidimensional scaling) and Adonis testing based on weighted UniFrac distance measure [31], [32]. An idealized tree for UniFrac analysis was computed from taxonomic assignments of each eOTU. We identified eOTUs that were significantly enriched in a sample category by applying a Welch-test individually for each eOTU across their abundances. The same test was applied for microbiome changes at family level, where abundances of each eOTU were summarized per taxonomic classification (family level) prior to significance testing. Data visualization was enhanced using the interactive tree of life [33].

#### Performance of microarray data for improved OTU calling

In this study, we used the well-established PhyloChip G3 DNA microarray for deciphering community relationships [25], [34]–[37]. Originally, microarray technology designed on the basis of a reference dataset of 16S rRNA genes does not allow the detection of precluded/unknown 16S rRNA genes when using a reference database for OTU calling [38], [39]. However, the approach used herein differs by the means of empirical OTU identification [28] and detected one eOTU affiliated to the SM1 Euryarchaeon (bootstrap 70%), although this archaeon has not been included in the original probe design of the array (method for classification of concatenated, interrupted probe sets was identical to method described below for 16S rRNA gene classification used in intergenic spacer region sequencing, see below). Consequently, this approach allowed inclusion of 16S rRNA genes not included in the chip design for microbial community relationship calculations. For details on empirical, non-supervised OTU calling the reader is referred to the Supplementary Information and Probst et al., 2014 [28].

#### SR-FTIR spectromicroscopy and data analysis

SR-FTIR spectromicroscopy using photon energy in the mid-infrared region (in frequency 4,000 to 650 cm^−1^, or in wavelength 2.5 µm to 15.4 µm, or in energy unit 0.496 eV to 0.081 eV, or 7.95×10^−20^ J to 1.29×10^−20^ J ; [40]) was used to obtain chemical information of the biofilm samples. Band assignment and spectra interpretation was done as described earlier [41]. More than 70,000 SR-FTIR spectra were collected for all biofilms at the infrared beamline (http://infrared.als.lbl.gov/) as described in Probst et. al 2013 [15]. For each spectrum, the ratio of the infrared absorbance from the membrane methyl (–CH_3_) group to that from the membrane methylene (–CH_2_) group was computed, and a threshold value of 0.75 was used to designate the spectrum to be archaeal (≥0.75) or bacterial (<0.75; [15]). Univariate analysis was used to obtain semi-quantitative information on sample biogeochemical composition (see Supplementary Information). For coupling infrared data with multivariate statistics aimed to profile the microbial communities, principal component-linear discriminant analysis (PCA-LDA) was performed in the Matlab (The MathWorks, Inc., Massachusetts USA) environment using lipid spectral window (2800–3100 cm^−1^) for archaeal communities, and lipids plus carbohydrates (1280-900 cm^−1^) for bacterial communities. Both datasets were vector normalized by the Amide II (1550±10 cm^−1^) absorption intensity.

#### Fluorescence *in situ* hybridization (FISH)

Whole-cell hybridization was carried out as described in earlier [14] with following probes (Rhodamine Green (RG) or CY3 labeled): EUB338/I [42], ARCH344 [12], SMARCH714 (SM1 Euryarchaeon; [12]) SRB385 [43] and Delta495a/b/c probe mix [44]. Bacterial positive controls (strain *Escherichia coli* K12, DSM 30083) and negative controls (non-sense probe NONEUB338) were used to validate the experiments. Thereafter the samples were analyzed as described by Probst et al. 2013 [15].

### Scanning electron microscopy (SEM) and transmission electron microscopy (TEM)

For SEM, drops of fixed samples (0.1% glutardialdehyde; w/v) were placed onto glass slides, covered with a coverslip, and rapidly frozen with liquid nitrogen. The coverslip was removed with a razor blade and the glass slide was immediately fixed with 2.5% (w/v) glutardialdehyde in fixative buffer, washed, postfixed with 1.0% osmium tetroxide, washed with buffer, followed by deionized water, dehydrated in a graded series of acetone solutions, and critical-point dried after transfer to liquid CO_2_. Specimens were mounted on stubs, coated with 3 nm of platinum using a magnetron sputter coater, and examined with a Zeiss Auriga scanning electron microscope operated at 1 kV. For TEM, fresh, unfixed biofilm pieces were deposited on a carbon-coated copper grid and negatively stained with 2% (w/v) uranyl acetate, pH 4.5 or 2.0% (w/v) phosphotungstic acid (PTA), pH 7.0. Samples were examined using a CM12 transmission electron microscope (Philips) operated at 120 keV.

#### Southern blotting of metagenomic DNA

Probes targeting the *hamus* gene were generated via amplification with the ham1f and ham2r primers (ham1f: 5′-CAGCATCAAAACAGGCGGGTGC-3′, ham2r: 5′-GTTCCTCTGAATTTGTATACGG-3′) and labeled with DIG High Prime as described in the manufacturer's instructions (Roche Diagnostics GmbH, Mannheim). 1 µg of metagenomic DNA of each biofilm type (Mühlbacher Schwefelquelle, Sippenauer Moor) was individually digested using the enzymes HincII and KpnI, then electrophoresed and blotted on a nylon membrane. After hybridization with the *hamus*-specific probe (in DIG Easy Hyb hybridization buffer incl. 50 ng DIG labeled probe, 40°C, 14 h), the membrane was blocked in TBST-B-buffer (Tris/HCl, pH 7.6 (20 mM); NaCl (0.8%, w/v), Tween 20 (0.1%, v/v), dry milk powder (5%, w/v)) followed by an antibody reaction with Anti-Digoxygenin-AP conjugate (dilution up to 1∶10000, Roche Diagnostics GmbH, Mannheim). The blot was washed thoroughly in 2× SSC (NaCl (0.3 M), trisodium citrate (0.03 M), pH 7.0) incl. 0.1% SDS at RT followed by a second washing step in 0.5× SSC incl. 0.1% SDS at 68°C. The detection was carried out through NBT/BCIP (nitro blue tetrazolium/5-bromo-4-chloro-3-indolyl-phosphate) reaction.

#### Intergenic spacer region sequencing of SM1 euryarchaeal strains

Parts of the archaeal rRNA gene operon were amplified from metagenomic biofilm DNA samples (MSI-BF and SM-BF) using 16S-345af (5′-CGGGGYGCASCAGGCGCGAA-3′ [45]) and 23S-64R (5′-GCCNRGGCTTATCGCAGCTT-3′ [46]). Amplicons were cloned in *E. coli* (TOPO TA cloning kit, TOP 10′ cells, Invitrogen) and 48 inserts per sample were bi-directionally sequenced (LGC Genomics, Berlin). Reverse sequences were trimmed to 16S rRNA genes and classified using the Naive Bayesian algorithm implemented in Mothur [47], [48] against an updated and 98%-clustered GreenGenes database (http://www.secondgenome.com/go/2011-greengenes-taxonomy/ [30]) supplemented with known archaeal 16S rRNA gene sequences from sulfidic springs. Sequences classified as SM1 Euryarchaea (bootstrap >90%) were individually assembled (forward and reverse read) and trimmed to the intergenic spacer region after multiple sequence alignments using MUSCLE [49]. Representative sequences of the intergenic spacer region were submitted to GenBank (accession numbers: KJ735447-9).

## Results

### Dominance of Archaea in subsurface biofilms confirmed by molecular approaches

**Figure 2:**
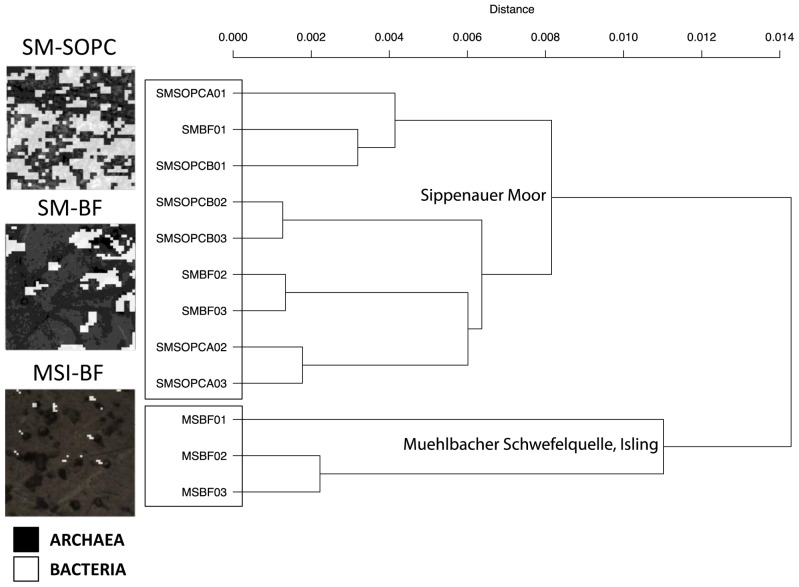
Abundance of Archaea and Bacteria in samples and the overall community relationship. Small panels present binary images of infrared data collected for three sample types, SM-BF (Sippenauer Moor, biofilm), SM-SOPC (Sippenauer Moor, string-of-pearls community) and MSI-BF (Mühlbacher Schwefelquelle, Isling, biofilm). Infrared maps show the distribution of Archaea and Bacteria in the samples. One pixel corresponds to 2 µm. Large panel: Hierarchical clustering based weighted UniFrac of abundance values of eOTUs (Bacteria and Archaea). Two different clusters separating the samples based on hydrogeology were observed.

PhyloChip G3, qPCR and FISH revealed the predominance of Archaea in the subsurface biofilms samples from Mühlbacher Schwefelquelle (MSI-BF) and Sippenauer Moor (SM-BF) samples (Table 1). QPCR showed that >97% of all 16S rRNA genes in MSI-BF and SM-BF samples were archaeal, but only 26% in the surface string-of-pearls community (SOPC). Cell counting after FISH staining showed 93% and 86% archaea in MSI-BF and SM-BF, respectively. These abundances were confirmed via SR-FTIR image analysis, which typically showed that archaea occupied >97% of the areas in MSI-BF and SM-BF, but only ∼38% in SOPC (Fig. 2, left panels).

**Table 1:** Quantification of archaeal and bacterial signatures via qPCR, FISH and SR-FTIR (values in brackes give standard deviation).

Method	Measurand	MSI-BF	SM-BF	SOPC
**QPCR (per ng DNA)**	Archaeal *16S rRNA* genes	2.89E+06 (±5.63E+05)*	2.09E+06 (±1.08E+06)	2.03E+05 (±5.48E+04)
	Bacterial *16S rRNA* genes	7.48E+04 (±7.65E+03)*	2.20E+04 (±2.68E+03)	5.75E+05 (±1.09E+05)
	*DsrB* genes	5.12E+03 (±7.87E+02)*	1.97E+03 (±9.82E+02)	3.46E+02 (±1.25E+02)
	Percent Archaea	97.44%*	98,96%	26,09%
**FISH**	Percent archaeal cells	92.96 (±2.16)	86.41 (±7.02)	ND
	Percent SRB385 stained Bacteria	85.4 (±4.7)*	39.32 (±11.81)	ND
	Percent DeltaMix stained Bacteria	89.2 (±0.9)*	63.87 (±14.70)	ND
**SR-FTIR**	Percent archaeal biomass	97.0 (±6.0)	97.1 (±4.4)	38.7 (±13.8)

*data from Probst et al., 2013.

ND: Not Determined.

### Site-specific microbiomes

PhyloChip G3 DNA microarray technology identified a total of 1300 bacterial and 37 archaeal eOTUs. An overview of all the different families detected in the samples is depicted in Fig. S2. Hierarchical analyses based on weighted UniFrac dissimilarities of all eOTUs revealed clusters of samples based on their geographical regions (SM versus MSI; Fig. 2, right panel), which is supported by a highly significant Adonis p-value (0.008). The macroscopic appearance of samples (BF vs. SOPC) was also identified to have a significant influence on the observed microbiome relationships (p-value = 0.003) for biofilm samples from both locations. Considering samples from SM only, the microbiome differences between SM-BF and SOPC were insignificant (p-value = 0.058) indicating a site-specific microbiome.

### Spatial dynamics of archaeome and bacteriome relationships

**Figure 3:**
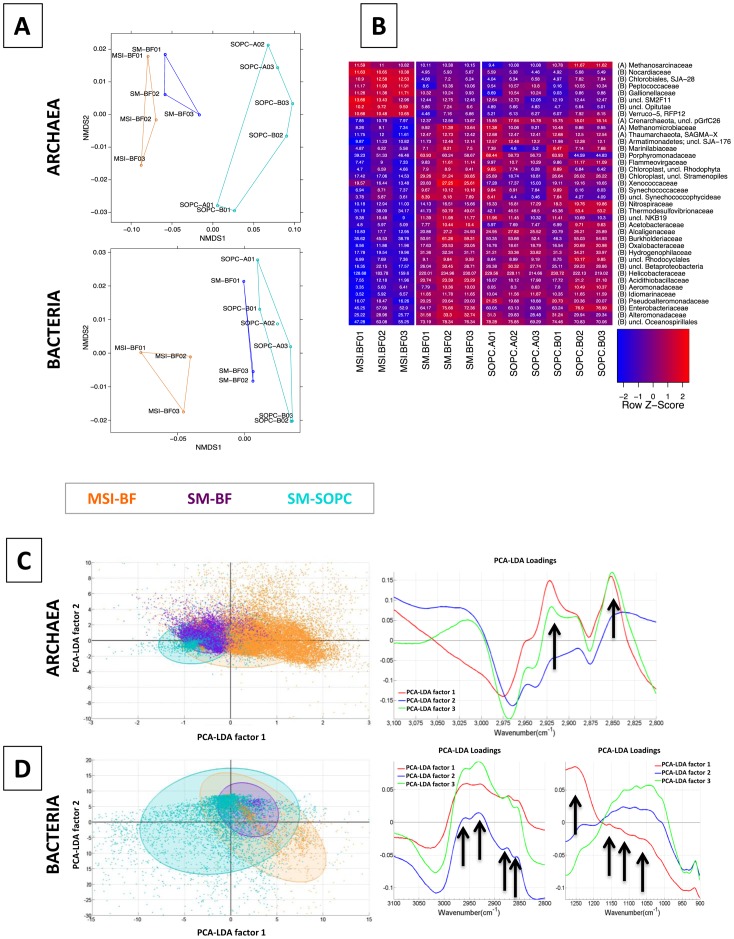
Detailed community profiling using PhyloChip G3 and SR-FTIR. A: Ordination analysis of PhyloChip G3 data based on weighted UniFrac measure of eOTU abundances followed by non-metric multidimensional scaling (NMDS). Stress for NMDS of archaeal eOTUs (#37): 0.0088. Stress for NMDS of bacterial eOTUs (#1300): 0.0223. B: Heatmap displaying significantly different families found between the two biofilm types, MSI-BF and SM-BF by PhyloChip G3 assay. Significance is based on aggregated HybScores of eOTUs on family level followed by a Welch-test. For false discovery detection please see Fig. S6. C: Ordination analysis of SR-FTIR data based on a linear discriminant analysis and principal component analysis (PCA-LDA) in the spectral region of 2800–3100 cm^−1^ on the archaea spectra extracted from the maps from the three different locations. On the right there is the plot of PCA-LDA loadings. PCA-LDA1 explains for the 93.4% of the variance, PCA-LDA2 for 5.3% and PCA-LDA3 for 0.9%. Arrows point to the infrared signals used to explain the difference between the samples: 2975 cm^−1^, 2965 cm^−1^, 2924 cm^−1^ and 2850 cm^−1^. D: PCA-LDA in the spectral regions of 900–1280 cm^−1^ and 2800–3100 cm^−1^ on SR-FTIR spectra of the bacteria “pixels” from the chemical maps of the samples at the three different locations. On the right there is a plot of PCA-LDA loadings in the two spectral region of interest. PCA-LDA1 explains for the 54.5% of the variance, PCA-LDA2 for 28.6% and PCA-LDA3 for 7.3%. Arrows point to the main infrared signals used to explain the difference between the samples: 2958 cm^−1^, 2925 cm^−1^, 2870 cm^−1^ and 2850 cm^−1^, in the second panel 1250 cm^−1^, 1110 cm^−1^, 1080 cm^−1^ and 1045 cm^−1^.

Aiming to analyze the microbial community relationships in detail, NMDS were performed on PhyloChip G3 derived 16S rRNA gene profiles of bacteria and archaea separately. For archaea, the NMDS plot (Fig. 3A, upper panel) separated MSI-BF and SM-BF from SM-SOPC along the NMDS1 axis. This implies a greater similarity in the archaeal community relationship among samples from the anoxic subsurface (biofilms) than among samples from same hydrogeological regions but of different oxygen content (SM-BF versus SOPC). These differences went along with an increased richness of archaeal eOTUs in SOPC samples, which also included e.g. Thaumarchaeota representatives. For Bacteria, however, the NMDS plot (Fig. 3A, lower panel) separated the MSI-BF samples from the SM-BF and the SM-SOPC samples, suggesting that the bacterial community relationship was affected more strongly by the sampling location and its hydrogeology (additional information in Table S2).

Notably, the bacterial composition of the Sippenauer Moor biofilm (SM-BF) and string-of-pearls community (SM-SOPC) appeared very similar (Fig. 3A, lower panel), although there was a strong increase in oxygen content from the biofilm sampling area to the SOPC sampling area. It might be concluded, that the bacteriome of the SM-BF tended to be maintained in the short travel distance to the SOPC (Fig. 1) along with oxygen increase.

### Both biofilms carried sulfate-reducing bacteria (SRB) with different taxonomic affiliation

290 of 1337 eOTUs were significantly different in their relative abundance when comparing MSI-BF samples with SM-BF samples (Fig. S3A, S4; p-values<0.05) resulting in separated microbiomes (Fig. S3B). We observed that eOTUs of certain phyla like Verrucomicrobia or Spirochaeta and two eOTUs classified as *Desulfobacteraceae* belonging to the twelve most significant eOTUs (Fig. S5) were significantly enriched in MSI-BF vs. SM-BF samples. Other members of this family of SRB were also significantly enriched in the SM-BF samples but with higher p-values [0.003∶0.050[.

Notably, SOPC samples clustered with SM-BF samples in hierarchal dendrograms based on the 290 Welch-test filtered taxa for biofilm communities (Fig. S3B) reflecting the similarity of these populations observed in other multivariate statistics mentioned earlier (Fig. 2, Table S2).

Considering summarized HybScores at family level (hybridization scores of eOTUs were aggregated across families based on taxonomic affiliation), 38 of 227 families had significant changes across aggregated abundances between biofilm categories (Fig. 3B, Fig. S6). The signatures of the designated SRB families like *Desulfobacteraceae*, *Desulfobulbaceae*, *Desulfovibrionaceae* did not show a significant variation between the MSI-BF and SM-BF (p-values were 0.37, 0.30, and 0.36, respectively). However, the abundance of *Desulfobacteraceae* displayed a significant difference between the two biofilms, MSI-BF/SM-BF, and the SOPC samples (p-value = 0.01). The eOTU and family level analysis allowed the conclusion that SM1 Euryarchaeon biofilms support an enrichment of members of SRB, with differences at eOTU level but similarities at their family level. In other words, SRB – which dominated the bacteriome based on FISH analyses – were members of the same family in the two biofilms but of different species or strains.

These data are in accordance with microscopic FISH data, and quantitative PCR of *dsrB* genes, which showed an increase of one order of magnitude in copy numbers for biofilm samples compared to SOPC samples (Table 1).

#### Similarity and variations of archaeal lipid signatures in biofilms

We applied PCA-LDA analysis to the SR-FTIR spectra previously categorized as archaeal or bacterial (Table 1) to gain insight into the biochemical differences in the composition at a functional group level of each microbiome. For the archaeal spectra, the two-dimensional PCA-LDA score plot revealed that the first PCA-LDA factor separated archaea in SM-BF and SM-SOPC samples from the majority (∼70%) of the archaea in the MSI-BF samples (Fig. 3C, left panel). The first loading vector (the red trace in Fig. 3C, right panel) showed that positive features near 2924 cm^−1^ and 2850 cm^−1^ were responsible for this separation. These frequencies correspond to the infrared absorption signals of the asymmetric and symmetric vibrations of CH_2_ in fatty acid chains of the membrane amphiphiles. Additional peaks at 2975-2965 cm^−1^ are associated to the methoxy CH stretching of –OCH_3_ and –OCH_2_ ethers [50]. Therefore, the PCA-LDA loadings plot suggested that the SM-BF and SM-SOPC archaea shared a similar membrane lipid composition, but differed from over 70% of the MSI-BF archaea. This could be explained by differences in the alkyl chain branching and in the polar heads [51]. Meanwhile, the two-dimensional PCA-LDA score plot of the bacterial spectra in both the lipid and the overall fingerprint region, were similar to microbiome relationships as revealed by PhyloChip G3 analysis (see above).

#### Ultrastructural differences exhibited in SM1-Euryarchaeon biofilms and hami appearance

A univariate analysis of the infrared absorption bands of the biomacromolecules (Fig. S7) confirmed that MSI-BF and SM-BF had the highest protein and lipid contents, whereas SM-SOPC the highest carbohydrate content. Consequently, samples from both biofilms were analyzed further using SEM and TEM to look into the ultrastructural differences.

**Figure 4:**
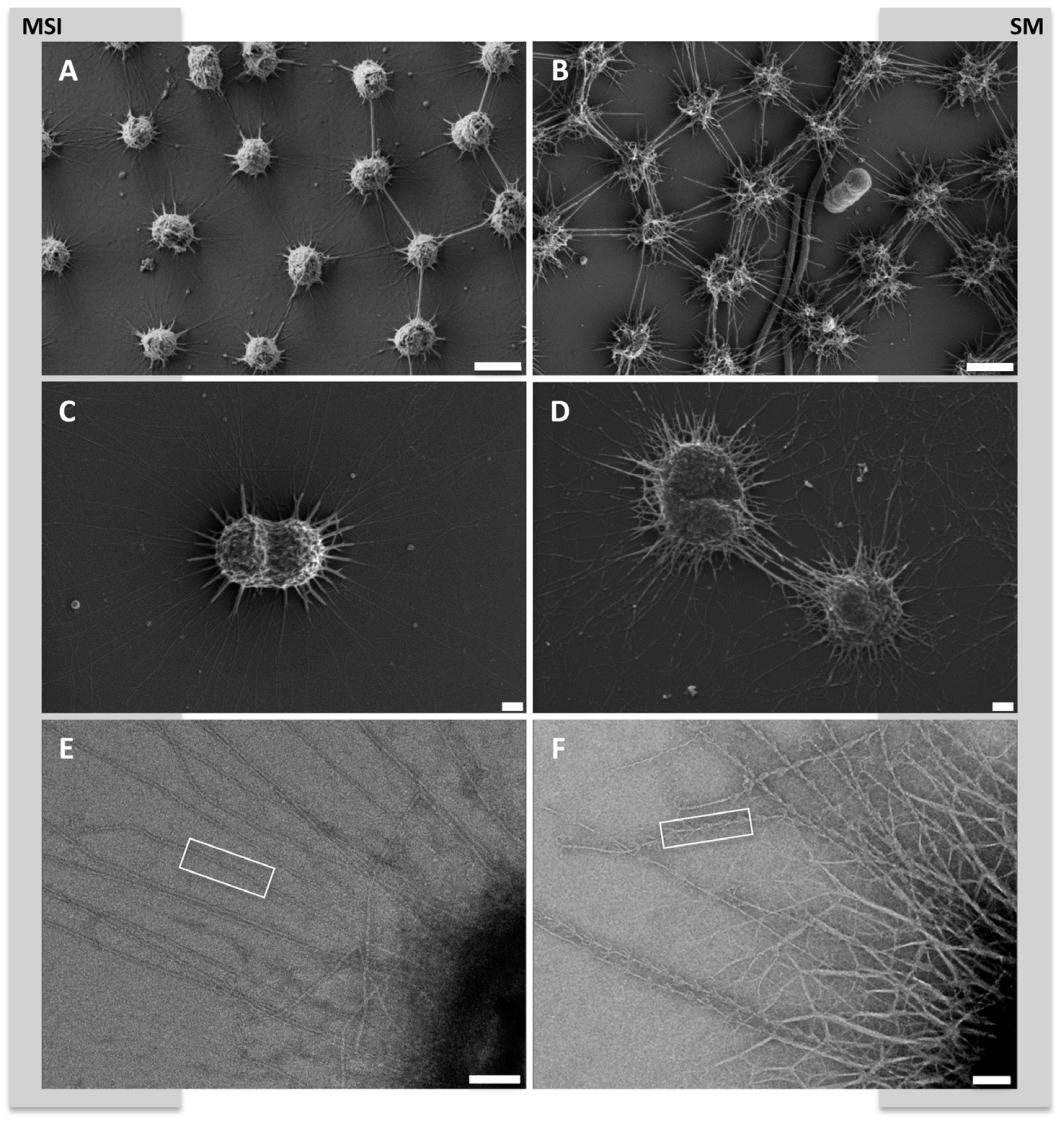
Scanning and transmission electron micrographs of biofilms, cells and hami. Left panels: MSI, right panel: SM. A: Scanning electron micrograph of MSI biofilm, showing SM1 euryarchaeal cells with defined distances and cell-cell connections. Bar: 1 µm. B: Scanning electron micrograph of SM biofilm, showing SM1 euryarchaeal cells with defined distances and fine-structured cell-cell connections. In-between: Bacterial filamentous and rod-shaped cells. Bar: 1 µm. C: Scanning electron micrograph of dividing SM1 euryarchaeal cell (MSI) with cell surface appendages. Bar: 200 nm. D: Scanning electron micrograph of dividing SM1 euryarchaeal cell (SM) with cell surface appendages. Bar: 200 nm. E: Transmission electron micrograph of cell surface appendages (hami) of SM1 euryarchaeal cells from the MSI biofilm. The hami carry the nano-grappling hooks, but besides that appear bare (square), without prickles (Moissl et al 2005). Bar: 100 nm. F: Transmission electron micrograph of cell surface appendages and matrix of SM1 euryarchaeal cells from the SM biofilm. The hami reveal the typical ultrastructure, with nano-grappling hooks and barbwire-like prickle region (square, Moissl et al 2005). Bar: 100 nm.

SM1 archaeal cells appeared as single or dividing cocci (Fig. 4), connected via a network of cell appendages and extracellular matrices. Considering one layer of SM1 Euryarchaea (Fig. 4A, 4B), most cells revealed regular distances to six neighboring cells in a hexagonal manner. Cells in the MSI biofilms were significantly larger than those in the SM biofilms (average diameter of 0.72 µm versus 0.60 µm, homoscedastic student's t-test of 40 cells each: p-value<10^−7^). Additionally, cell surfaces were napped and connections were smoother in MSI biofilms (Fig. 4C), whereas cell surfaces in the SM biofilms appeared fluffy with more connections between cells (Fig. 4D).

Cells in both biofilms carried hami with distal hooks that appeared correctly folded (Fig. 4E–F). Nevertheless, SM1 Euryarchaea in the MSI biofilm revealed only a low percentage of such correctly folded hami structures with respect to the ‘prickle region’ [17]. Prickles seemed absent in most MSI hami (Fig. 4E), whereas such “bare” hami were sparsely observed for SM biofilm cells (Fig. 4F).

#### Two strains of the same archaeal species dominated the two biofilms

It was believed that the SM1 Euryarchaeota from SM and MSI were identical based on analysis at the 16S rRNA gene level [18]. Yet both showed such strong variations in the membrane lipid composition (Fig. 3C) and ultrastructure (Fig. 4), implicating possible differences between the two archaeal populations at genomic level. Under this observation a comparative Southern blot analysis of biofilm DNA (MSI versus SM) with probes specifically designed to target the *hamus* gene, that encodes for the major protein of the unique cell surface appendages, was performed [17]. Using different restriction enzymes (HincII and KpnI) hybridization signals of several distinct bands were retrieved (Fig. S8). This result indicated at least the presence of more than one *hamus* gene in both samples. Moreover, there is a reliable difference in the restriction pattern between the metagenomic DNA from both biofilm types. Notably, SM1 cells purified from the SM-SOPC [12] produced the same pattern as SM-BF. Additionally, sequencing of 96 clones of intergenic spacer regions (between 16S rRNA gene and 23S rRNA gene), without pre-selection of the clones via RFLP [18], showed that six single nucleotide polymorphisms existed between the two dominant sequences from the MSI-BF and SM-BF (Fig. S9), while the 16S rRNA gene sequences were identical providing evidence for different dominant strains of SM1 Euryarchaeota at the two sampling sites.

## Discussion

Subsurface microbial life exists in an environment that is challenging in many ways: lack of sunlight, mostly cold temperatures, low nutrient levels and often anoxic conditions demand alternative ways of carbon assimilation and energy production. This includes the usage of other electron acceptors than oxygen, resulting in anaerobic respiration or fermentation [52], [53]. To date, information on subsurface life is very limited. This is either due to the restricted accessibility of subsurface biotopes or due to the detection of many unexplored microbial taxa therein, which remain uncultivated and thus largely not understood [4], [5], [54]. In this regard, the two vicinal sulfidic springs studied here provide a stable and well accessible window to the subsurface and allow the exploration and comparison of archaeal biofilms delivered to the surface. While microbiome profiling on 16S rRNA gene sequences revealed similar archaeomes, the SR-FTIR approach uncovered striking differences in archaeal lipid signatures at a molecular level. These variations were either caused by the presence of different organisms (strains of the same archaeal species) or by altered gene expression of the same organism, most likely reflecting adaptive responses to different environmental conditions. Meanwhile, PhyloChip data of the two biofilms revealed the enrichment of designated sulfate-reducing bacteria (SRB) of different taxonomic affiliation at OTU but not at family level, which, however, seemed to share widely diverse lipid compositions as revealed by SR-FTIR ordination.

The constant co-appearance and bacterial predominance of such potentially sulfate-reducing deltaproteobacteria within both SM1 biofilms suggests a possible syntrophic relationship [15], [16]. Even more, the presence of sulfate-reducers could point to environmental conditions prevailing in the biofilms' original biotopes and thus the growth conditions of the SM1 Euryarchaeon.

Generally, SRB's sulfate-reducing activity is linked to the oxidation of organic compounds or molecular hydrogen and to the formation of H_2_S, an important biogenic compound found in considerable amount (0.85 mg/l) in both spring waters. As a requirement for the SRB catalyzed reactions, the biotope, or respective environment, needs to fulfill at least the following criteria: a) anoxic conditions, b) sulfate as an electron acceptor, and c) an electron donor, most likely either organic molecules or hydrogen. It could therefore be hypothesized, that the SM1 Euryarchaeon thrives under these conditions or even provides such an environment, creating a convenient biotope for SRB.

**Figure 5:**
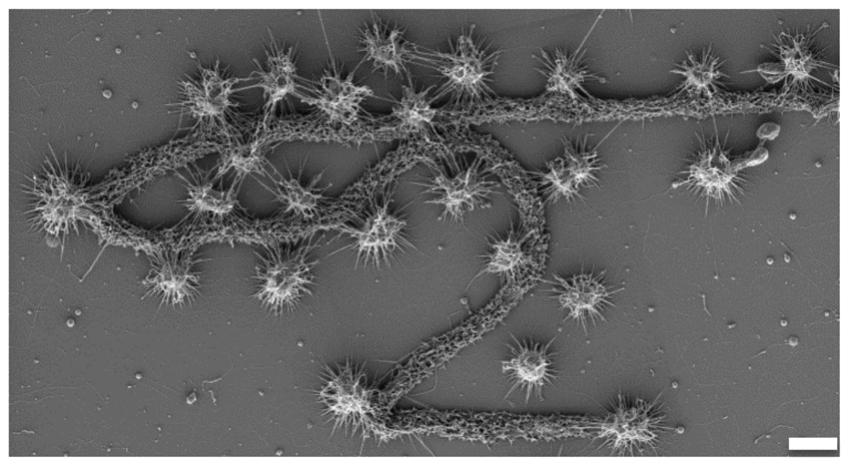
Scanning electron micrograph of filamentous bacterium and surrounded and cocooned by the SM1 euryarchaeal cells (SM-BF). Bar: 1 µm.

When biofilm pieces are washed up to oxygen-mixing areas of the surface spring water and attach to rigid material, the entire community is transformed into a string-of-pearls-like macroscopic appearance. The archaeal diversity increased, as shown for instance by the detection of Thaumarchaeota. This observation is supported by SR-FTIR analyses, which revealed that in both SM-BF and MSI-BF the amount of proteins and lipids is higher in respect to SM-SOPC. Consequently, the anaerobic biofilm frameworks were richer in proteins potentially accounting for the abundance of *hami* proteins, whereas the a polysaccharide-rich framework is promoted in the SOPC. The bacteria originally being part of the biofilm, are also absorbed into the string-of-pearls community, as the ratio of archaeal 16S rRNA genes and *dsrB* genes remains almost constant throughout all samples indicating a potential key role of sulfate-reducing bacteria in SM1 Euryarchaeon communities. This process of the formation of the string-of-pearls community is completed by the most likely directed settling of filamentous, sulfide-oxidzing bacteria (*Thiothrix*, *Sulfuricurvum*), which cover the archaeal microcolony and become an equal partner of the SM1 Euryarchaeon [11], [13]. Supporting evidence for this hypothesis comes from the fact that filamentous bacteria were cocooned by cell surface appendages of SM1 Euryarchaeota in biofilm samples (Fig. 5) and similar bacteriomes were found for the biofilm and the SOPC at the Sippenauer Moor. The biofilm microbiome consequently persists and can therefore be considered precursors of the string-of-pearls community (Fig. 1).

Based on hydrogeology we can exclude a direct exchange of biomaterial between both aquifer outlets with respect to the subsurface water current (both aquifers are artesian). Additionally, even though both wells are fed by the same deep groundwater flow within the pre-alpine Tertiary Molasse basin, a parallel transport of microbial communities from these regions to both springs, Sippenauer Moor and Mühlbacher Schwefelquelle, can be excluded: If delivered simultaneously to both biotopes from the same origin, one would expect similar patterns in (bio-)geochemical profiles and microbial diversity, since the biofilms analyzed were sampled in parallel (within one day). It is assumed that the local hydrogeology and geochemistry in the subsurface of the individual springs are responsible for creating different biotopes and causing differences in the observed microbiome structure. Chemical composition analysis of both spring waters and microscale infrared imaging of areas rich in archaeal signatures did not reveal obvious differences in concentration or presence of certain compounds and thus could not explain the divergence of the archaeal communities. However, the two springs have one major difference: the MSI spring was drilled (depth of the spring well almost 30 m [15]), whereas the SM spring is a natural spring. A drilled bore hole surely provides different conditions including a straight, quicker water flow, compared to a natural water flow, which might be much more labyrinthine and slower. Future comparative genomic and transcriptomic studies may reveal the potential niche differentiation of the archaea at the two springs.

Although a number of details with respect to archaeal 16S rRNA gene sequences, prevalence of SRB and general biofilm-structure are in agreement, the communities from both locations, and also the archaea themselves, reveal severe differences at various levels. For instance, SR-FTIR detected location dependent shifts in lipid profiles of biofilm associated archaea. In general, lipid variations can be growth phase dependent [55], point to a specific biotope-adaptation [56], [57] and thus reflect influences from environmental parameters in both biotopes – or simply mirror strain-specific properties. The latter possibility is supported by detectable differences in fingerprint experiments with metagenomic DNA and within the archaeal SM and MSI 16S-23S rRNA gene intergenic spacer regions. Consequently, Southern-blotting and intergenic spacer analysis, together with the above-mentioned SR-FTIR analysis, and ultrastructural analyses suggested that two different SM1 euryarchaeal populations dominate the biofilms that can be found at the Mühlbacher Schwefelquelle and at the Sippenauer Moor. To our knowledge, this is the first report of a natural divergence of one archaeal species in nature, which can potentially be attributed to niche differentiation in these two biotopes.

Studying these archaeal communities, which still remain dark matter with regard to biochemical cycling, provided insight into the local impact of the biotope on microbiome variation and into potential microbial niche differentiation. Our multifarious results, based on the commingling of established and novel methods, have added another piece to the puzzle in order to understand the dynamics of subsurface microbial life in such a great, dark and little explored environment.

## Supporting Information

Figure S1 **Geographical map of sampling locations.**
(PDF)

Figure S2 **Entire microbiome.**
(PDF)

Figure S3 **Significantly different eOTUs in biofilm samples.**
(PDF)

Figure S4 **Testing for false discovery detection of eOTUs.**
(PDF)

Figure S5 **Significantly different eOTUs in biofilm samples: The top 12.**
(PDF)

Figure S6 **Testing for false discovery detection of families.**
(PDF)

Figure S7 **Univariate analyses of SR-FTIR absorption band ratios.**
(PDF)

Figure S8 **Southern blot analysis.**
(PDF)

Figure S9 **Alignment of intergenic spacer regions.**
(PDF)

Table S1 **Chemical water analysis.**
(PDF)

Table S2 **Multivariate statistics of microbiome data.**
(PDF)

Methods S1 **Microbiome profiling using PhyloChip G3.**
(PDF)
