# Supplementary material for: Coupling Genetic and Chemical Microbiome Profiling Reveals Heterogeneity of Archaeome and Bacteriome in Subsurface Biofilms That Are Dominated by the Same Archaeal Species
Source: PLoS One. 2014 Jun 27;9(6):e99801. doi: 10.1371/journal.pone.0099801 (PMC4074051; doi:10.1371/journal.pone.0099801)

Figure S1: Geographical map illustrating the sampling locations Sippenauer Moor (SM, 1) and Muehlbacher Schwefelquelle, Isling (MSI, 1) in Germany and in their relative position to Regensburg and the Danube (“Donau”).

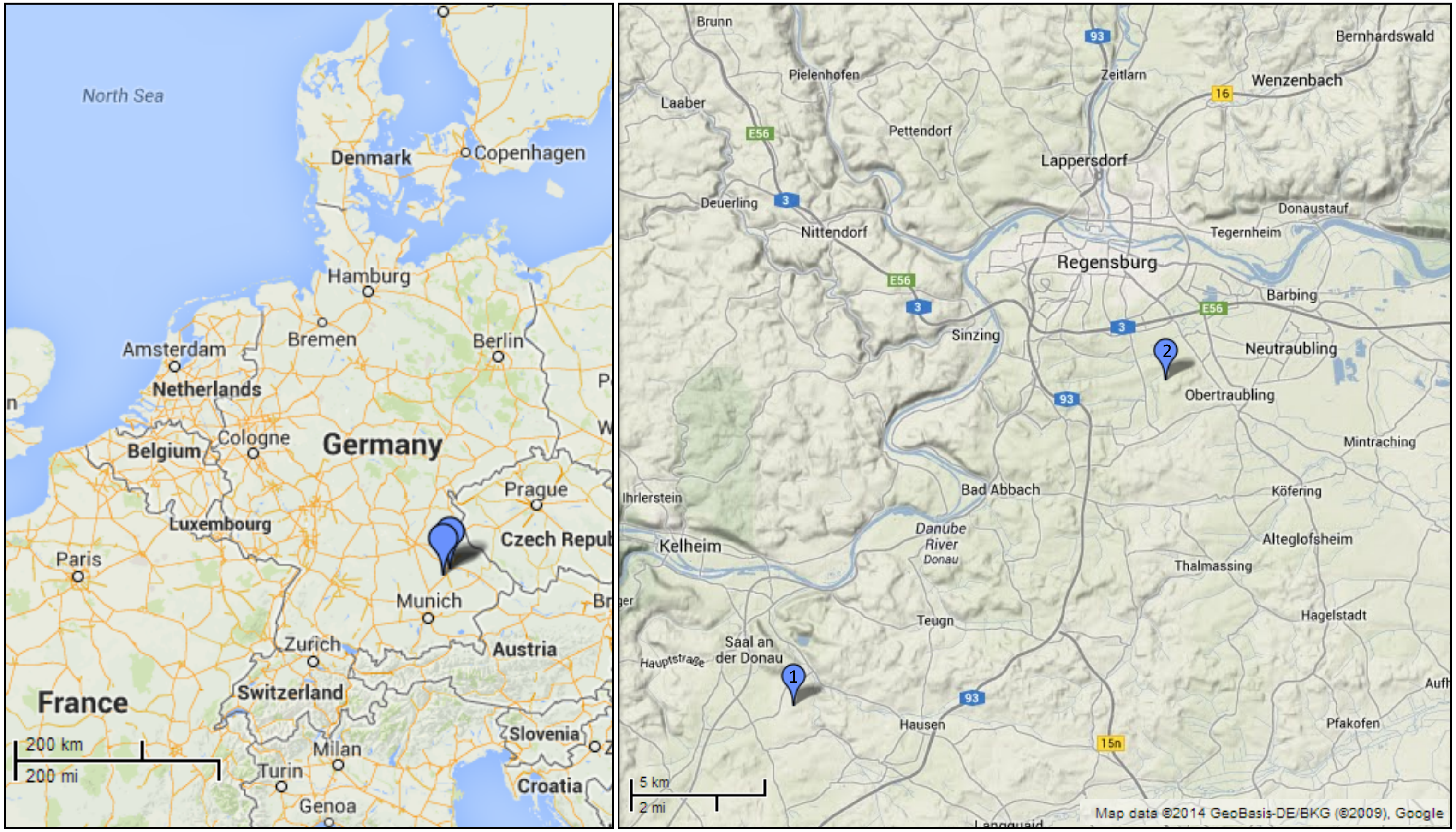

Supplement: Figure S1 — Geographical map of sampling locations. (PDF) [file pone.0099801.s001.pdf]
