# Supplementary material for: Coupling Genetic and Chemical Microbiome Profiling Reveals Heterogeneity of Archaeome and Bacteriome in Subsurface Biofilms That Are Dominated by the Same Archaeal Species
Source: PLoS One. 2014 Jun 27;9(6):e99801. doi: 10.1371/journal.pone.0099801 (PMC4074051; doi:10.1371/journal.pone.0099801)

Figure S2: Overall microbiome detected across all samples. For each family detected in the microbiome one eOTU with the most drastic change in abundance from biofilm to string-of-pearls community was selected. In case eOTUs within one family behaved non-uniformly, two eOTUs with the most drastic increase and decrease in abundance were selected. HybScores were normalized as z-scores individually for each eOTU across all samples. An idealized tree based on taxonomic assignments was computed and OTUs belonging to the nine major phyla were colored differently from other phyla that are colored in petrol.

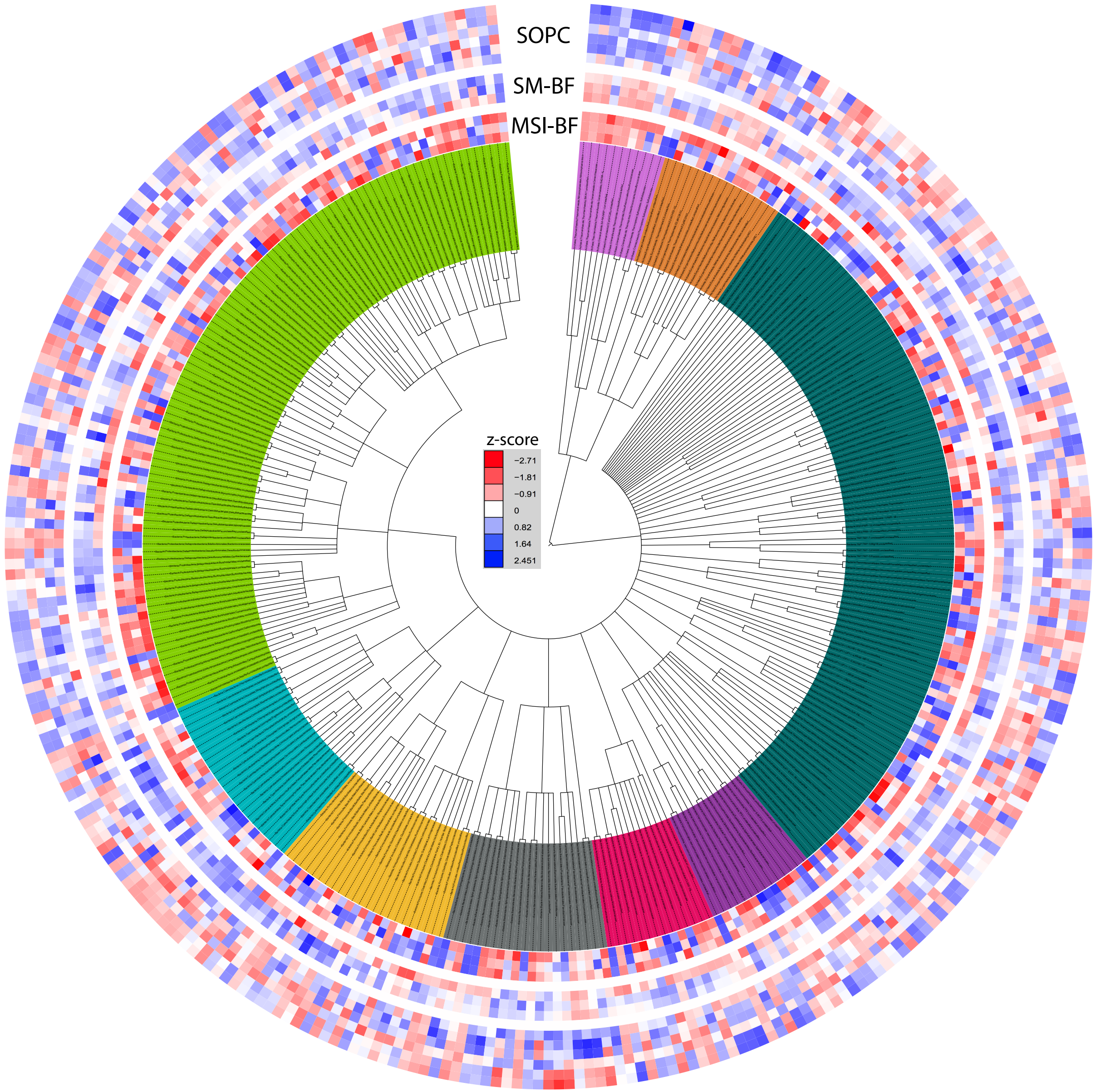

Supplement: Figure S2 — Entire microbiome. (PDF) [file pone.0099801.s002.pdf]
