# Supplementary material for: Coupling Genetic and Chemical Microbiome Profiling Reveals Heterogeneity of Archaeome and Bacteriome in Subsurface Biofilms That Are Dominated by the Same Archaeal Species
Source: PLoS One. 2014 Jun 27;9(6):e99801. doi: 10.1371/journal.pone.0099801 (PMC4074051; doi:10.1371/journal.pone.0099801)

Figure S3: EOTUs found to be significantly different between the two biofilm samples (#290) by a Welch test. **A**: Heatmap displaying the 290 eOTUs. Value = HybScore. For false discovery analysis due to multiple testing the reader is referred to Figure S3. **B**: Hierarchical clustering based on average neighbour (weighted UniFrac of 290 eOTUs) showing a separation of the microbiomes based on geographic location. Interestingly, SOPC samples which were not subject to significance filtering group together with SM-BF samples indicating similar microbiomes.

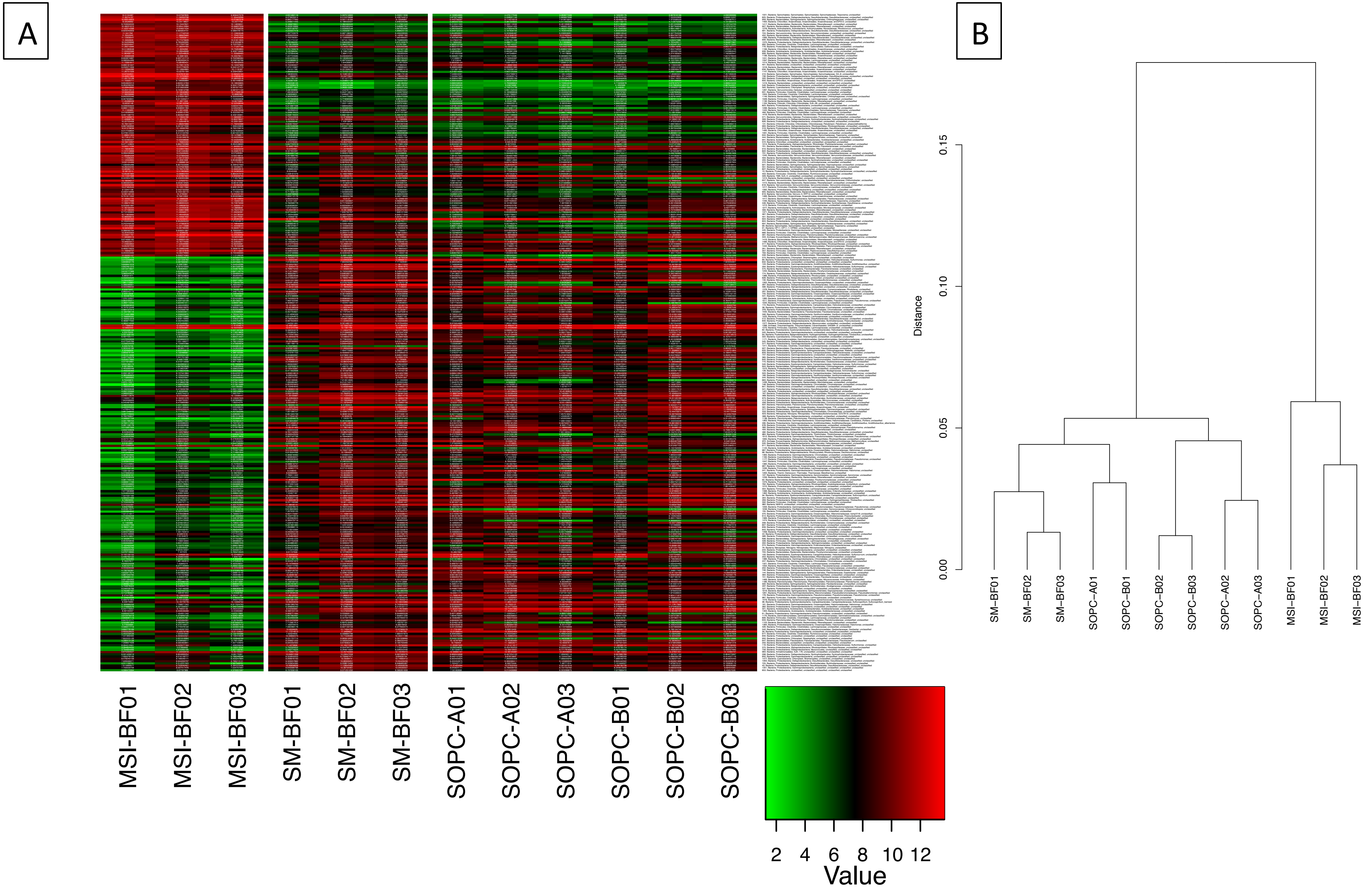

Supplement: Figure S3 — Significantly different eOTUs in biofilm samples. (PDF) [file pone.0099801.s003.pdf]
