# Supplementary material for: Coupling Genetic and Chemical Microbiome Profiling Reveals Heterogeneity of Archaeome and Bacteriome in Subsurface Biofilms That Are Dominated by the Same Archaeal Species
Source: PLoS One. 2014 Jun 27;9(6):e99801. doi: 10.1371/journal.pone.0099801 (PMC4074051; doi:10.1371/journal.pone.0099801)

Figure S4: Testing for false discovery detection of eOTUs. Permutation of sample group assignments for the Welch-test. In 100% of 100 permutations, equal or less than 290 eOTUs were identified. In approximately 20% of all permutations 290 eOTUs were identified. These 20% are expected since the possibility of creating the initial grouping after permutation is 20%. Consequently, the possibility of finding 290 eOTUs is unlikely due to chance. Red = true value. Y-axis displays the number of taxa passing the Welch-test.

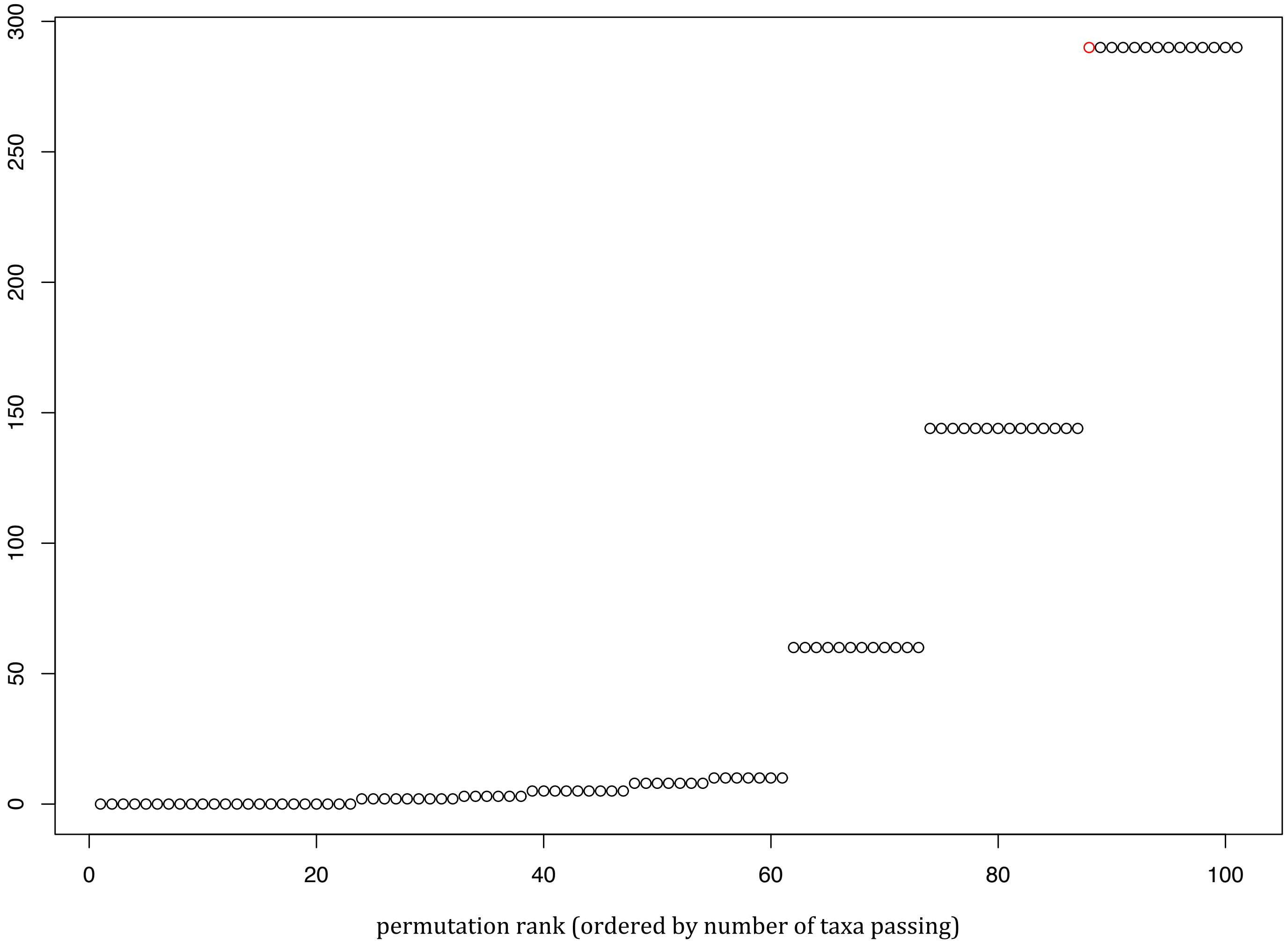

Supplement: Figure S4 — Testing for false discovery detection of eOTUs. (PDF) [file pone.0099801.s004.pdf]
