# Supplementary material for: Coupling Genetic and Chemical Microbiome Profiling Reveals Heterogeneity of Archaeome and Bacteriome in Subsurface Biofilms That Are Dominated by the Same Archaeal Species
Source: PLoS One. 2014 Jun 27;9(6):e99801. doi: 10.1371/journal.pone.0099801 (PMC4074051; doi:10.1371/journal.pone.0099801)

Figure S5: Differences of the two biofilm-microbiomes: Top 12 of the most significant taxa found between the two biofilm types MSI-BF and SM-BF (Welch test; for details see Fig. S2).

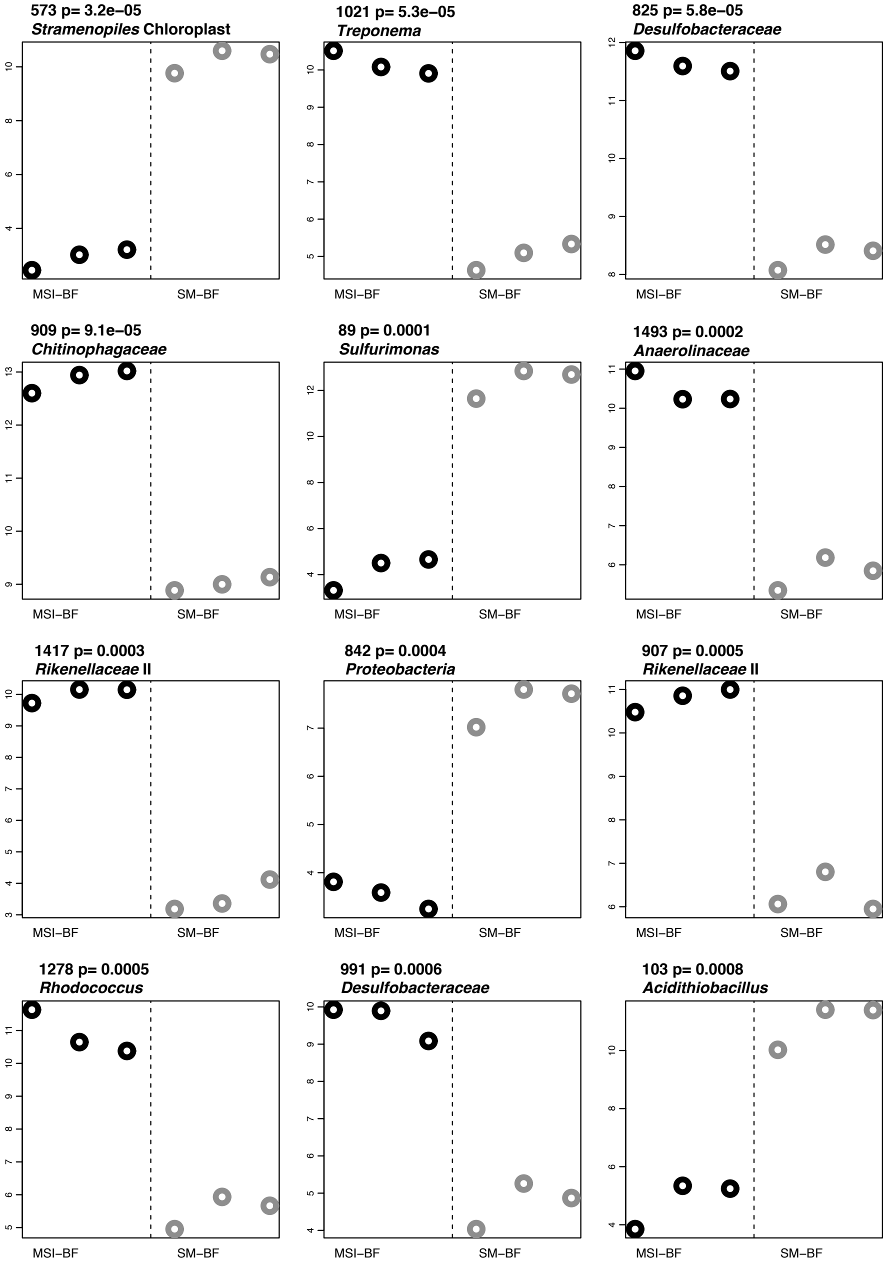

Supplement: Figure S5 — Significantly different eOTUs in biofilm samples: The top 12. (PDF) [file pone.0099801.s005.pdf]
