# Supplementary material for: Coupling Genetic and Chemical Microbiome Profiling Reveals Heterogeneity of Archaeome and Bacteriome in Subsurface Biofilms That Are Dominated by the Same Archaeal Species
Source: PLoS One. 2014 Jun 27;9(6):e99801. doi: 10.1371/journal.pone.0099801 (PMC4074051; doi:10.1371/journal.pone.0099801)

Figure S6: Testing for false discovery detection of aggregated hybscores of families (Figure 3B). Permutation of sample group assignments for the Welch-test. In approximately 5% of 100 permutations, more than 38 families passed the Welch test with a p-value lower than 0.05. Consequently, the possibility of finding 38 families is unlikely due to chance. Red = true value. Y-axis displays the number of taxa passing the Welch-test.

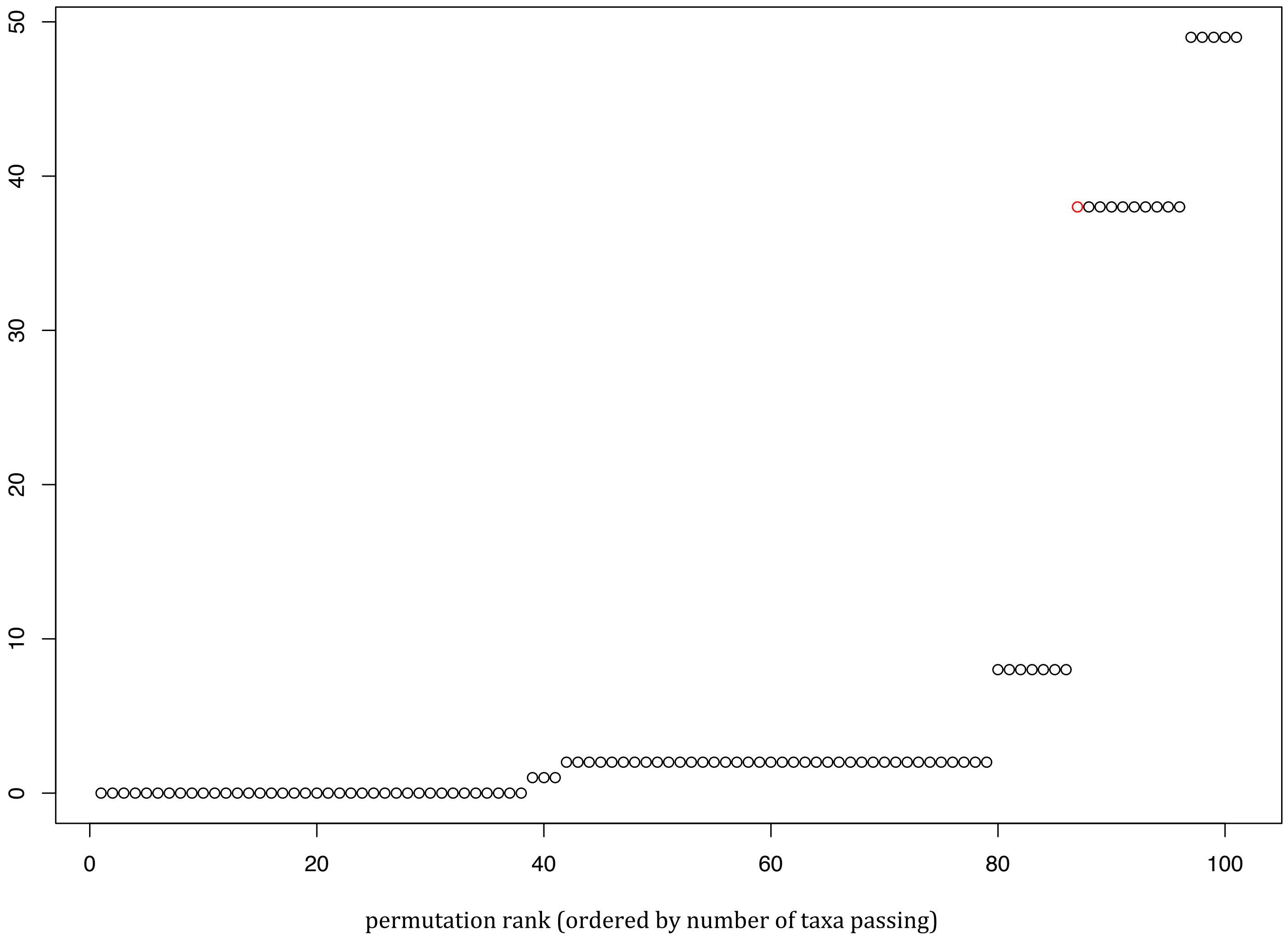

Supplement: Figure S6 — Testing for false discovery detection of families. (PDF) [file pone.0099801.s006.pdf]
