# Supplementary material for: Coupling Genetic and Chemical Microbiome Profiling Reveals Heterogeneity of Archaeome and Bacteriome in Subsurface Biofilms That Are Dominated by the Same Archaeal Species
Source: PLoS One. 2014 Jun 27;9(6):e99801. doi: 10.1371/journal.pone.0099801 (PMC4074051; doi:10.1371/journal.pone.0099801)

Figure S7: Univariate analyses of SR-FTIR absorption band ratios. A-B) The comparison of total proteins and lipids to the carbohydrate amount can provide information on the overall composition of the matrix that composes these communities; we can observe that in both SM-BF and MSI-BF the amount of proteins and lipids is higher in respect to SM-SOPC, implying that the anaerobic biofilm frameworks are richer in proteins and in our case can accounts for the abundance of *hami* proteins, whereas the aerobic environment of the SOPC promotes a polysaccharide rich framework. C-D) Evaluation of nitrogen and sulfur compounds shed light on the metabolic imprints in the immediate geochemical surroundings at the time of sampling. A comparison of the ratio of NO_2^- to NO_3^- (NO_2^-/NO_3^-) and the ratio of S^{2-} to SO_4^{2-} (S^{2-}/SO_4^{2-}). These analyses revealed spatial variations in the microbial reduction/oxidation processes.

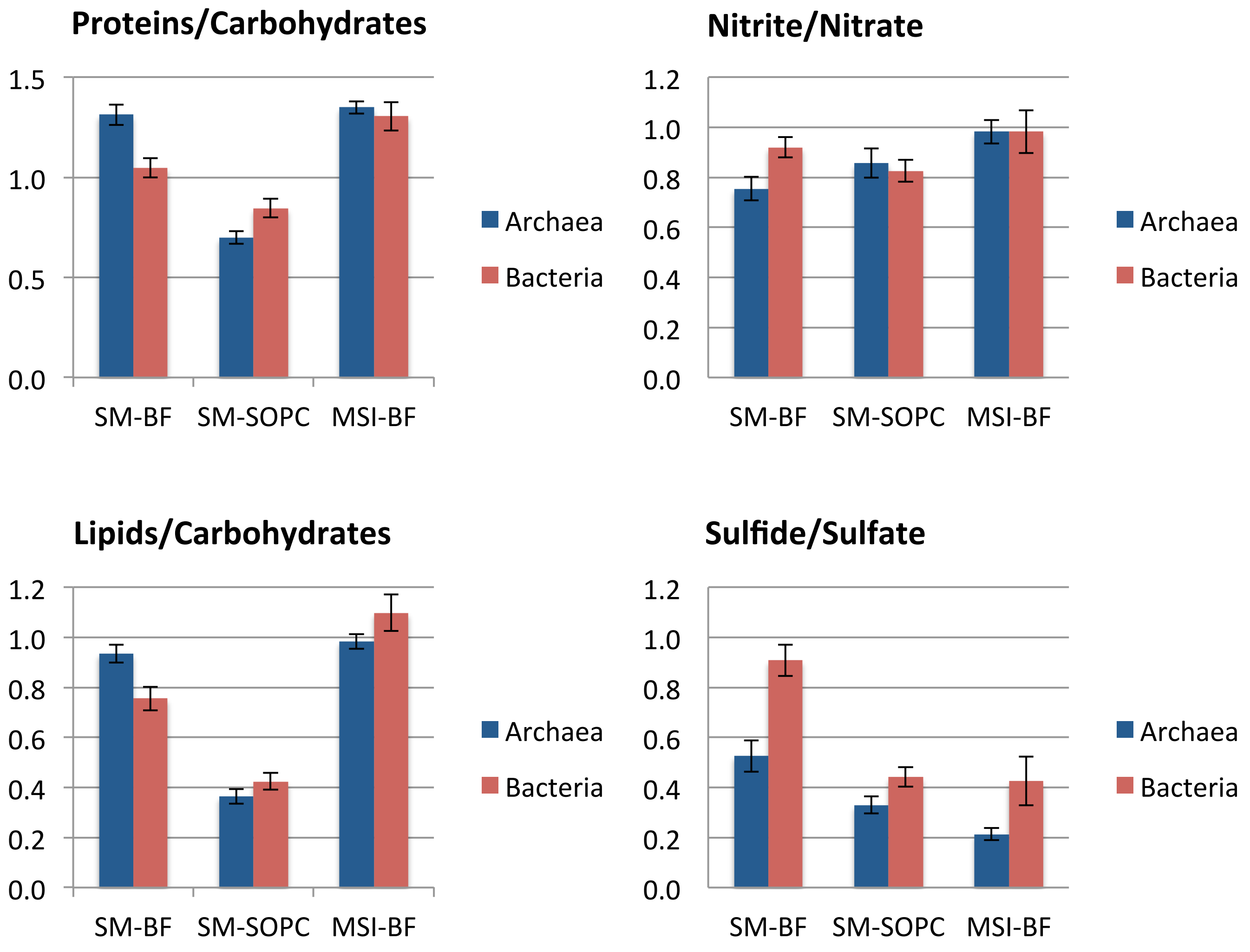

Supplement: Figure S7 — Univariate analyses of SR-FTIR absorption band ratios. (PDF) [file pone.0099801.s007.pdf]
