# Supplementary material for: Coupling Genetic and Chemical Microbiome Profiling Reveals Heterogeneity of Archaeome and Bacteriome in Subsurface Biofilms That Are Dominated by the Same Archaeal Species
Source: PLoS One. 2014 Jun 27;9(6):e99801. doi: 10.1371/journal.pone.0099801 (PMC4074051; doi:10.1371/journal.pone.0099801)

Figure S8: Southern blot analysis of metagenomic DNA collected from two sampling sites where the SM1 Euryarchaeon occurs. A different restriction pattern was observed for metagenomic DNA from the two sampling sites indicating a difference of SM1 Euryarchaea on genome level. Remarkably, the same pattern was retrieved for the string-of-pearl-community as it was retrieved for the biofilm from the same sampling site. This supports the hypothesis of the biofilm being the precursor of the SOPC.

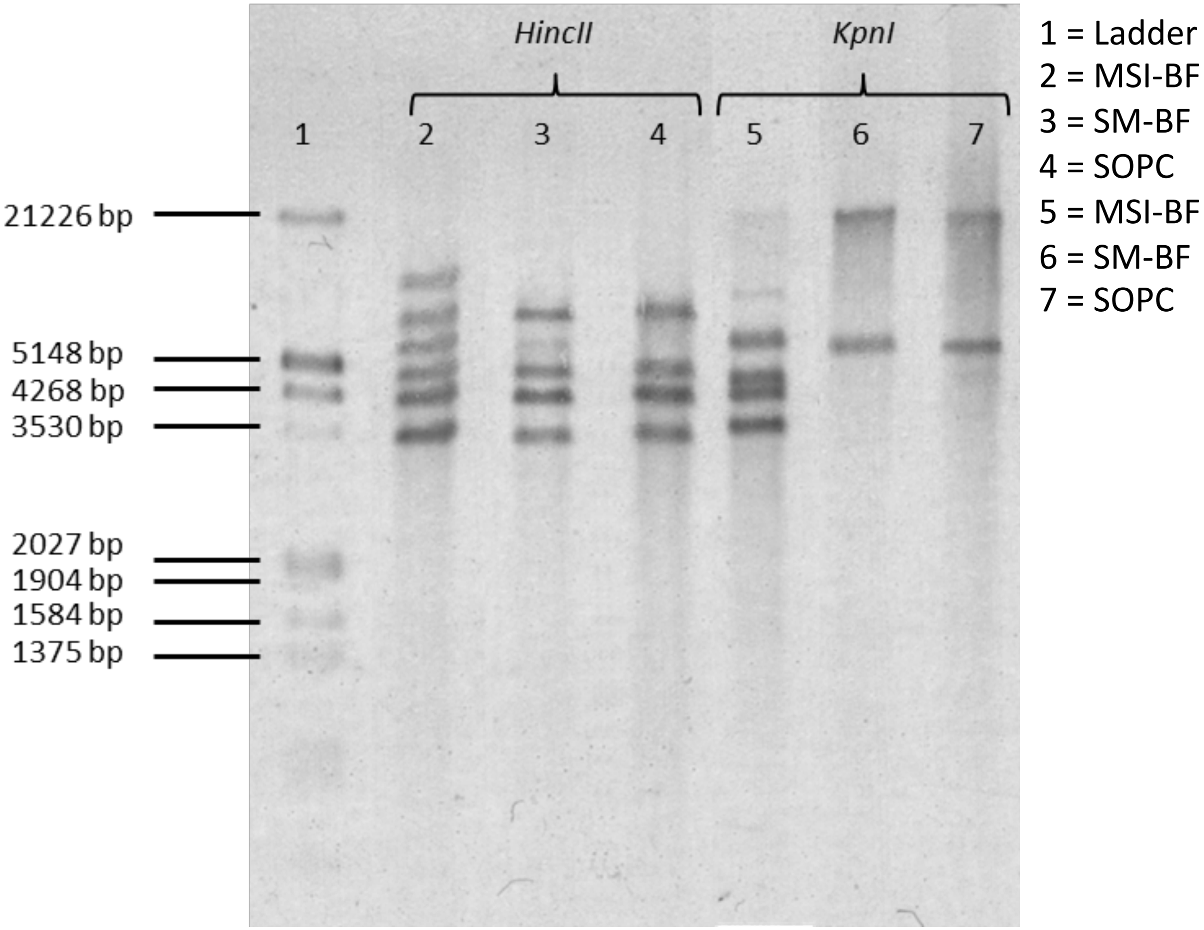

Supplement: Figure S8 — Southern blot analysis. (PDF) [file pone.0099801.s008.pdf]
