# Supplementary material for: Coupling Genetic and Chemical Microbiome Profiling Reveals Heterogeneity of Archaeome and Bacteriome in Subsurface Biofilms That Are Dominated by the Same Archaeal Species
Source: PLoS One. 2014 Jun 27;9(6):e99801. doi: 10.1371/journal.pone.0099801 (PMC4074051; doi:10.1371/journal.pone.0099801)
Supplement: Table S1 — Chemical water analysis. (PDF) [file pone.0099801.s010.pdf]

Table S1: Analysis of chemical compounds measured in spring waters from Sippenauer Moor (SM) and Mühlbacher Schwefelquelle (MSI). Data taken from Rudolph et al., 2004.

Chemical compound	SM (compound in mg/l)	MSI (compound in mg/l)
SO_4^{2-}	31	16
$S_2O_3^{2-}$	16	14
SO_3^{-}	<1	<1
NO_3^{-}	<1	<1
NO_2^{-}	<0.02	<0.02
NH_4^{+}	0.13	0.33
Na^{+}	14	21
Cl^{-}	13	22
PO_4^{3-}	<0.05	<0.05
Ca^{2+}	67	79
Mg^{2+}	20	27
K^{+}	3.0	6.1
Mn	0.019	0.081
Ni	<0.01	<0.01
As	<0.002	<0.002
Al	<0.05	<0.05
Sb	<0.003	<0.003
B	0.10	0.13
Cu	<0.005	<0.005
Se	<0.003	<0.003
Zn	<0.05	<0.05
Fe	0.025	0.021
CO_2, dissolved	19	32
