# Supplementary material for: Coupling Genetic and Chemical Microbiome Profiling Reveals Heterogeneity of Archaeome and Bacteriome in Subsurface Biofilms That Are Dominated by the Same Archaeal Species
Source: PLoS One. 2014 Jun 27;9(6):e99801. doi: 10.1371/journal.pone.0099801 (PMC4074051; doi:10.1371/journal.pone.0099801)
Supplement: Table S2 — Multivariate statistics of microbiome data. (PDF) [file pone.0099801.s011.pdf]

Table S2: Overview of multivariate statistics performed on the bacterial microbiome and on the archaeal microbiome. Entire microbiome is used for finding significantly eOTUs between sample groups (Welch-test). "+" = positive, "-" negative HC-AN refers to hierarchial clustering based on average neighbour.

**Factor**		**Hydrogeological location**	**Appearence**	**Groups**	**SM**	**MSI-SOPC**	**Biofilms**
**Bins**		MSI | SM	BF | SOPC	MSI-BF | SM-BF | SOPC	SM-BF | SOPC	MSI-BF | SOPC	MSI-BF | SM-BF
**Sample counts**		3 | 9	6 | 6	3 | 3 | 6	3 | 6	3 | 6	3 | 3
**Bacterial microbiome**	**NMDS distinct**	+	-	-	-	+	+
	**HC-AN distinct**	+	-	-	-	+	+
**Archaeal microbiome**	**NMDS distinct**	-	+	-	+	+	-
	**HC-AN distinct**	-	-	-	-	-	-
**Entire microbiome**	**Diff. eOTU count**	512	480	522	248	550	290
