# Supplementary material for: Coupling Genetic and Chemical Microbiome Profiling Reveals Heterogeneity of Archaeome and Bacteriome in Subsurface Biofilms That Are Dominated by the Same Archaeal Species
Source: PLoS One. 2014 Jun 27;9(6):e99801. doi: 10.1371/journal.pone.0099801 (PMC4074051; doi:10.1371/journal.pone.0099801)

Figure S9: Alignment of intergenic spacer regions of the SM1 Euryarchaon population from MSI-BF and SM-BF, which occurred at least twice in the analysis. Singletons are omitted, only 8 sequences per samples are given. The majority of MSI-BF sequences showed six SNPs compared to the majority of SM-BF sequences. Numbers give positions in the 375 character alignment. Accession numbers are KJ735447-9.

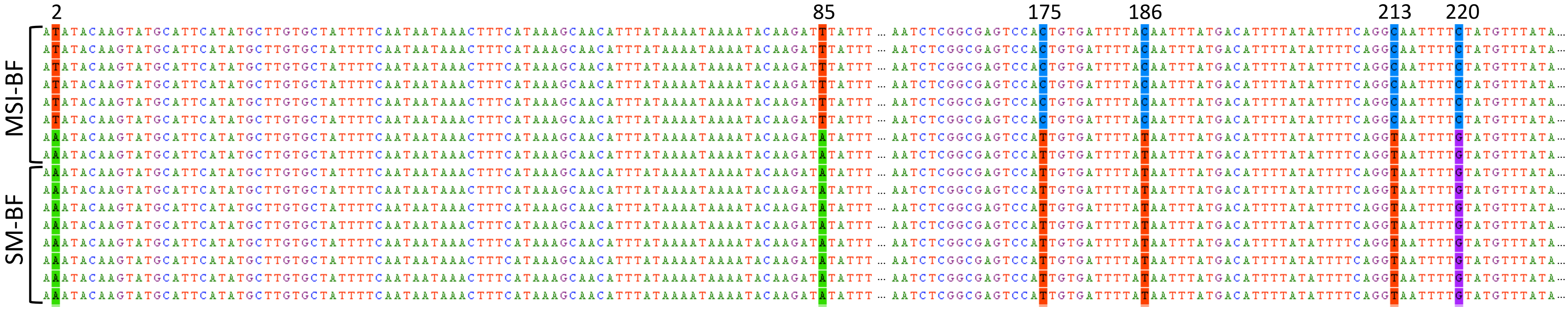

Supplement: Figure S9 — Alignment of intergenic spacer regions. (PDF) [file pone.0099801.s009.pdf]
