# Supplementary material for: Coupling Genetic and Chemical Microbiome Profiling Reveals Heterogeneity of Archaeome and Bacteriome in Subsurface Biofilms That Are Dominated by the Same Archaeal Species
Source: PLoS One. 2014 Jun 27;9(6):e99801. doi: 10.1371/journal.pone.0099801 (PMC4074051; doi:10.1371/journal.pone.0099801)
Supplement: Methods S1 — Microbiome profiling using PhyloChip G3. (PDF) [file pone.0099801.s012.pdf]

Supplemental Methods S1: Microbiome Profiling using PhyloChip G3 [28]
Amplified 16S rRNA genes from each sample were hybridized to G3 PhyloChip™ arrays (Second Genome, San Bruno, CA), which display 1,016,064 oligonucleotide probes capable of tracking microbial population shifts across all known bacterial and archaeal taxa, from both culturable and not-yet culturable organisms originally discovered in animal, industrial, environmental, and clinical specimens including mammalian gut specimens. Individual probes are mapped to the Greengenes 16S rRNA gene database [29] using the 2012 taxonomy [30]. The hybridization procedures have been described in previous work [25, 39].
To calculate the summary fluorescence intensity (FI) for each probe feature on each array, the central nine pixels of individual image features were ranked by intensity and the 75th percentile was used. Probe FIs were background-subtracted and scaled to the PhyloChip™ Control Mix [35]. Array FI is collected as integer values ranging from 0 to 65,536 (2^{16}). Fluorescence intensity observed from perfectly matching (PM) probes were compared to mis-matching (MM) probes and were considered positive if PM/MM >= 1.5 and PM-MM >= 100*N and r >= 0.95 where N indicates the array specific noise, and r represents the response score [25, 35]. In total, the FI values for 25,154 PM probes passed in at least 3 samples were exported from all experiments. FI values were then $\log_2$ transformed and used as input to empirical probe-set discovery where individual probes were clustered into probe-sets based on both 1) correlations in ranked FI across all samples and 2) taxonomic relatedness. Where multiple clustering solutions were available, higher correlation coefficients were favored over lower correlation coefficients, taxonomic relatedness at the species level was favored over higher ranks, and sets composed of a greater number of probes were favored over those with fewer numbers. All probe sets contained >= 5 probes with average pair-wise correlation coefficients >= 0.85. A total of 1,380 probe-sets were found and the empirical operational taxonomic units (eOTU) tracked by each probe-set were taxonomically annotated against Greengenes using the combination of the 9-mers contained in all probes of the set. Bayesian scoring was applied and the taxonomic resolution was constrained to the level (i.e. order, family, genus) where >80% bootstrapped confidence was achieved. The Bayesian annotation, as opposed to adopting the annotation from full-length reference sequences, prevents over-specification of the eOTU. The mean ranked FI among the multiple probes for each eOTU was calculated for each sample. These values are referred to as the hybridization score (HybScore) used in PhyloChip abundance-based analysis.
